# A Facile Method to Control Pore Structure of PVDF/SiO_2_ Composite Membranes for Efficient Oil/Water Purification

**DOI:** 10.3390/membranes11110803

**Published:** 2021-10-22

**Authors:** Qianqian Xu, Yuchao Chen, Tonghu Xiao, Xing Yang

**Affiliations:** 1 School of Materials Science and Chemical Engineering, Ningbo University, Ningbo 315211, China; 1911072040@nbu.edu.cn (Q.X.); 2011086016@nbu.edu.cn (Y.C.); 2 Department of Chemical Engineering, KU Leuven, Celestijnenlaan 200F, B-3001 Leuven, Belgium

**Keywords:** PVDF/SiO_2_ composite membrane, lower critical solution temperature (LCST), nonsolvent thermally induced phase separation (NTIPS), structural control, oil-in-water emulsion

## Abstract

The use of poly(vinylidene fluoride) (PVDF) microfiltration (MF) membranes to purify oily water has received much attention. However, it is challenging to obtain high-performance PVDF microfiltration membranes due to severe surface fouling and rapid decline of permeability. This study explored a new approach to fabricate high-performance PVDF/silica (SiO_2_) composite membrane via the use of a polymer solution featuring lower critical solution temperature (LCST) characteristics and the non-solvent thermally induced phase separation method (NTIPS). Coupling with morphological observations, the membrane formation kinetics were analyzed in depth to understand the synergistic effect between the LCST solution properties and fabrication conditions in NTIPS. Utilizing such a synergistic effect, the transition from finger-like macrovoid pores to bi-continuous highly connected pores could be flexibly tuned by increasing the PVDF concentration and the weight ratio of SiO_2_/PVDF in the dope solution and by raising the coagulation temperature to above the LCST of the solution. The filtration experiments with surfactant-stabilized oil-water emulsion showed that the permeation flux of the PVDF/SiO_2_ composite membranes was higher than 318 L·m^−2^·h^−1^·bar^−1^ and the rejection above 99.2%. It was also shown that the PVDF/SiO_2_ composite membranes, especially those fabricated above the LCST, demonstrated better hydrophilicity, which resulted in significant enhancement in the anti-fouling properties for oil/water emulsion separation. Compared to the benchmark pure PVDF membrane in oily water purification, the optimal composite membrane T70 was demonstrated via the 3-cycle filtration experiments with a significantly improved flux recovery ratio (*F_rr_*) and minimal reduced irreversible fouling (*R_ir_*). Overall, with the developed method in this work, facile procedure to tune the membrane morphology and pore structure was demonstrated, resulting in high performance composite membranes suitable for oil/water emulsion separation.

## 1. Introduction

The discharge of large amounts of oily wastewater from various sources (e.g., oil and gas, food and beverage, maritime, textile, and machining) not only pose significant challenges to our environment, but also cause water pollution affecting human health [1,2,3,4]. The loss of expensive oil products is also considered as an economic drawback. Amongst all forms of oil/water, namely, free-floating, un-emulsified oil, and emulsified oil, the emulsified oil is the most difficult one to purify by conventional physical or chemical separation methods due to the stabilized oil droplets at the submicron to micron size range, such as settling, flotation, hydrocyclone, fenton process, and coagulation. Alternatively, membrane-based separation is considered highly efficient and cost-effective for removing oil droplets smaller than ~10 μm and potentially recovering them for reuse [3,4,5,6]. There are several advantages to using membranes, such as the production of high-quality effluent, a small footprint, scalable processing and automation, and reduced chemical and energy consumption. In particular, microfiltration (MF) membranes have been widely investigated for treating oil/water emulsion [4,7,8].

Poly(vinylidene fluoride) (PVDF) is one of the most versatile membrane materials because of its superior thermal and chemical stability, resistance to γ-radiation, and excellent biocompatibility. It has been widely used in the fabrication of microfiltration membranes for water treatment [9,10,11]. Particularly, the use of PVDF MF membranes to separate the oil phase from emulsified water has received great attention [12,13]. Phase inversion methods are commonly adopted to fabricate PVDF MF membranes, such as nonsolvent induced phase separation (NIPS), thermally induced phase separation (TIPS), and nonsolvent thermally induced phase separation (NTIPS, i.e., combined NIPS and TIPS) [11,14,15,16]. The phase separation mechanism of the NIPS method is induced by exchange between nonsolvent and solvent, whereas for TIPS, the phase separation mechanism is induced by a temperature change of the dope solution. The polymer dope solution used in the TIPS process can be classified into two types, namely the upper critical solution temperature (UCST) and lower critical solution temperature (LCST) systems, respectively [11,17,18]. It is noted that most of these studies of PVDF polymer solution were featured as UCST characteristics. Recently, we developed a quaternary PVDF/polyvinylpyrrolidone (PVP)/*N,N*-dimethylacetimide (DMAc)/magnesium chloride (MgCl_2_) dope solution system that featured a LCST characteristic, resulting in the development of a new type PVDF membrane with highly connected pore structure via the NTIPS method. It was demonstrated that the developed NTIPS PVDF membrane showed superior performance in oily water purification, simultaneously achieving high permeability and high solute rejection to overcome the classical trade-off relationship in general membrane processes [11].

However, one of the main obstacles that limits the long-lasting performance of PVDF MF membranes is severe membrane fouling in oily water treatment and eventual loss of performance due to its inherently hydrophobic nature [19,20,21]. To reduce the fouling effect, membrane researchers have made substantial efforts in developing surface modification methods to enhance the hydrophilicity and oleophobicity of PVDF membranes [21,22,23]. These modification methods are generally classified as chemical grafting and physical blending. Physical blending, particularly by incorporating hydrophilic inorganic nanoparticles (e.g., silica (SiO_2_), titanium dioxide) into the PVDF membrane matrix has gained considerable attention because of the simplicity of preparation by the phase separation method in one single step and effectiveness in improving the anti-fouling performance. Among these inorganic nanoparticles, SiO_2_ nanoparticles are widely adopted due to their good hydrophilicity, stability, low cost, and compatibility with the organic solvents used in PVDF dope solutions [23,24]. Liao et al. fabricated PVDF/SiO_2_ composite ultrafiltration membranes. The results showed that the PVDF/SiO_2_ membranes exhibited a higher water flux and improved anti-fouling performance compared with the pure PVDF membranes [25,26]. Yu et al. reported that the hydrophilicity and permeability of PVDF/SiO_2_ composite hollow fiber membranes was improved [24]. Several similar studies reported that the main performance controlling factor in PVDF/SiO_2_ composite membrane fabrication is the homogeneous distribution of inorganic nanoparticles within the membrane matrix, which is closely related to the resultant membrane characteristics such as hydrophilicity and pore structure [27,28,29,30]. Thus far in the literature the preparation of PVDF/SiO_2_ composite membranes via blending mainly utilized the UCST PVDF solution systems with either TIPS or NIPS methods.

In this study, we explored a new approach to fabricate high-performance PVDF/SiO_2_ composite membranes via the use of a polymer solution featuring LCST characteristics. The membrane formation kinetics were analyzed via the synergistic effect between the LCST solution properties and fabrication conditions in non-solvent thermally induced phase separation (NTIPS). The influence of the SiO_2_ addition on the pore structure formation and membrane performance was investigated. First, the LCST behavior of PVDF/SiO_2_ dope solutions at varying PVDF concentrations and weight ratios of SiO_2_ were studied. Second, the membrane morphologies were investigated in terms of the effects of PVDF concentration, weight ratio of SiO_2_/PVDF, and coagulation temperature. The feasibility to tune the pore structure from finger-like macrovoids to cellular-like bi-continuous pores was examined. Third, the separation experiments of synthetic oily water were conducted to evaluate the membrane performance in terms of the rejection properties, permeability, and response to oil fouling. Finally, the resistance to oil fouling of the membrane was also investigated to evaluate its suitability for oily water purification.

## 2. Experimental

### 2.1. Materials and Chemicals

PVDF was purchased from Solvay (Shanghai) Co., Ltd., China (model: 1015). The polyvinylpyrrolidone (PVP, model: K30) and hydrophilic silica nanoparticles (SiO_2_, 7–40 nm) were purchased from Aladdin Reagent Inc., Shanghai, China. The *N,N*-dimethylacetimide (DMAc), anhydrous magnesium chloride (MgCl_2_), sodium dodecyl sulfate (SDS), ethanol, and isopropanol (IPA) were purchased from Sinopharm Reagent Inc., China. The DMAc, MgCl_2_, SDS, ethanol, and IPA were analytical grade and used as received.

### 2.2. Characterization of PVDF/SiO_2_ Dope Solutions

#### 2.2.1. Solution Preparation

The PVDF/SiO_2_ dope solution is composed of five components: PVDF, PVP, SiO_2_, DMAc, and MgCl_2_. First, MgCl_2_ was completely dissolved in the DMAc solvent, then pre-determined amounts of SiO_2_ were added into the DMAc/MgCl_2_ solution and stirred to ensure a homogeneous dispersion. The PVDF and PVP were then dissolved into the aforementioned solution and stirred at 30 °C to form a homogeneous dope solution. A series of PVDF/SiO_2_ dope solutions (labeled from M1 to M6) of various compositions were prepared to evaluate the effect of PVDF concentration, weight ratio of SiO_2_/PVDF, and coagulation temperature on the structure and properties of these membranes, as listed in Table 1. For comparison, the neat PVDF dope solution without SiO_2_ was labeled as M0 to fabricate the control membrane.

#### 2.2.2. Cloud Points

The cloud point (T_cloud_) of the dope solution is defined as the LCST in this study, which corresponds to the phase separation temperature of the dope solution induced by temperature change. The T_cloud_ was observed by using an in-house made light transmittance device based on our previously reported method [11].

### 2.3. Preparation of PVDF/SiO_2_ Composite Membranes

The homogeneous PVDF/SiO_2_ dope solutions listed in Table 1 were degassed before casting. Then the solutions were uniformly cast onto glass plates by an automated casting machine described in our previously report [31]. The nascent membranes were subsequently immersed into a water coagulation bath at a pre-determined temperature (30–90 °C). Finally, to completely remove the solvent and additive, the wet membranes were kept in fresh deionized water at room temperature.

### 2.4. Membrane Characterization

The membrane surface and cross-sectional structure were observed by using field emission scanning electron microscopy (S-4800, Hitachi, Japan). The membrane samples were immersed and subsequently fractured in liquid nitrogen. The sample was then coated with platinum by an ion sputtering device. The Si elemental analysis of SiO_2_ nanoparticles distributed in the PVDF/SiO_2_ composite membranes was performed by energy dispersive X-ray (EDX) spectroscopy (APOLLO XL, AMETEK, Inc., Berwyn, PA, USA). The contact angle measurement on the top surface of the membrane was conducted using a goniometer (DSA 100, DKSH group). The light transmittance of the nascent membranes formed in the coagulation bath was measured by the optical device as described in our previously report [11]. The mechanical properties of the membranes were measured using an electronic universal testing machine (Shenzhen Kaiqiangli Testing Instruments Co. Ltd., Shenzhen, China, model: KDIII-0.05). The membrane porosity (*ε*) was defined as the ratio of the pore volume to the total volume of the membrane, and the detailed measurement process and calculation method can be found from the previously report [11]. Five samples were measured to get average data of the mechanical and porosity properties.

The mean pore radius (*r*_m_) was determined according to the filtration velocity method and calculated by the Guerout-Elford-Ferry equation. The maximum pore radius (*r*_max_) was measured by the bubble point method with an in-house-built porometer and calculated by Laplace’s equation based on the bubble point pressure, which was described elsewhere in the literature [4,32,33]. The value of *r*_max_/*r*_m_ can be used as an index to distinguish the distribution curves of the pore size, i.e., narrow or wide [33].

### 2.5. Membrane Performance Testing

#### 2.5.1. Preparation of Oil-in-Water Emulsion

A surfactant-stabilized emulsion with an oil concentration of 5000 mg/L, which was prepared by dissolving 5 g soybean oil and 0.5 g sodium dodecyl sulfate (SDS) in 1 L deionized water, was used as a model feed to evaluate the antifouling properties of the membranes. To obtain a stable and homogenous oil-in-water emulsion, the mixture was sonicated for 20 min and stirred at 420 r/min for 9 h. The oil droplet size distribution of the emulsion was measured using a Mastersizer analyzer (Nana ZS 90, Malvern Instrument, Worcestershire, UK).

#### 2.5.2. Filtration Performance with Pure Water and Oil/Water Emulsion

The membrane fluxes of pure water and oil-in-water emulsion were measured by a filter cup (Shanghai Mosu Science Equipment Co. Ltd., Shanghai, China, model: MSC300) with a dead-end filtration mode at a pressure of 20 kPa. The effective membrane area was 35 × 10^−4^ m^2^. The permeated fluxes of pure water (*Jw*) and oil-in-water emulsion (*Jp*) were calculated by Equation (1):

*J* = *V*/(*A* · *t* · Δ*P*) (1)

where *J* is the permeated water flux of the membrane (L·m^−2^·h^−1^·bar^−1^), *V* is the volume of permeated water (L), *A* is the effective membrane area (m^2^), *t* is the filtration time (h), and Δ*P* is the operation pressure (bar^−1^).

The oil concentration of the feed and permeate solutions can be quantified by the light absorbance of these solutions, measured by an ultraviolet spectrophotometer (UV-Vis) (Beijing Presee Instruments Co. Ltd., Beijing, China, model: TU-1900). The membrane rejection *R* of oil droplets can be calculated by Equation (2):

*R* = (1 − *C_p_*/*C_f_*) × 100% (2)

where *C_p_* and *C_f_* represent the oil content calculated by the absorbance of the permeate and feed solutions, respectively.

#### 2.5.3. Fouling Evaluation Experiments

The oil/water emulsion prepared as shown in Section 2.5.1 was used as a model feed to evaluate the antifouling properties of the membranes. The fouling evaluation experiment process was continuously conducted in three cycles and each cycle contained the following steps. First, the initial pure water flux (*J*_*w*1_) of the membrane was measured with deionized water at 20 kPa for a 30-min period. Second, the model feed was used as the feed and the filtration test was performed for 1 h at 20 kPa with stirring. The permeate flux (*J_f_*) of the membrane was recorded. After filtration of the model feed solutions, the contaminated side of the membrane was cleaned in-situ via clean water rinsing. Finally, to evaluate the recovery of flux after filtration of the emulsion, the pure water flux (*J*_*w*2_) of the cleaned membrane was measured again with deionized water for 30 min. It should be noted that the last step of the previous cycle was also the first step of the next cycle. The antifouling properties of the membrane in each cycle can be evaluated by the calculated fouling indicators, namely, water flux recovery ratio (*F_rr_*), the total fouling ratio (*R_t_*), the reversible fouling ratio (*R_r_*), and the irreversible fouling ratio (*R_ir_*) [11,34]. The water flux recovery ratio (*F_rr_*) was calculated by Equation (3):

*F_rr_* = *J_w_*_2_/*J_w_*_1_ × 100% (3)

where *J*_*w*1_ and *J*_*w*2_ are defined as the pure water flux of the fresh membrane and used membrane (after cleaning), respectively.

The fouling indexes such as total fouling ratio (*R_t_*), the reversible fouling ratio (*R_r_*), and the irreversible fouling ratio (*R_ir_*) are quantified via the following equations:

*R_t_* = (*J_w_*_1_ − *J_f_*)/*J_w_*_1_ × 100% (4)

*R_r_* = (*J_w_*_2_ − *J_f_*)/*J_w_*_1_ × 100% (5)

*R_ir_* = (*J_w_*_1_ − *J_w_*_2_)/*J_w_*_1_ × 100% (6)

where *J_f_* is defined as the permeation flux (L·m^−2^·h^−1^·bar^−1^) at the emulsion filtration step.

## 3. Results and Discussion

### 3.1. The Cloud Point of PVDF/SiO_2_ Dope Solutions

**Figure 1:**
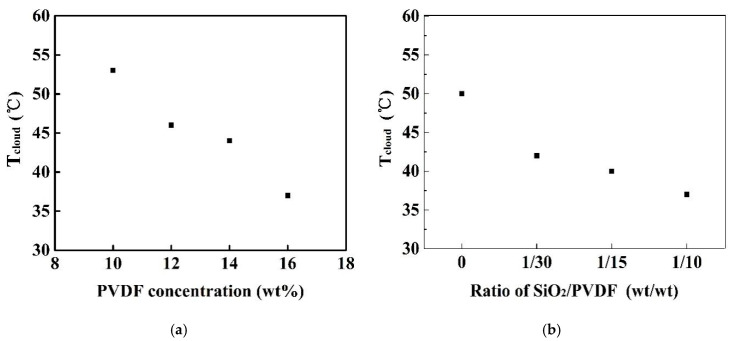
Effect of PVDF concentration and the weight ratio of SiO_2_/PVDF on the cloud point of PVDF/SiO_2_ dope solutions. (**a**) effect of PVDF concentration. (**b**) effect of the weight ratio of SiO_2_/PVDF.

The quaternary PVDF/MgCl_2_/PVP/DMAc solution has LCST characteristics, and its mechanism was reported in our previously published work [11]. In this study, the LCST characteristics of the dope solution with SiO_2_ added to the above quaternary solution system was investigated and the effect of the weight ratio of SiO_2_/PVDF and PVDF concentration of the dope solutions on the LCST were studied. Figure 1 illustrates the cloud points (T_cloud_) of the dope solutions listed in Table 1. The cloud points of the dope solutions M3 to M6 at the same weight ratio (1:10) of SiO_2_/PVDF showed an increasing trend from 37 °C to 53 °C as the concentration of PVDF decreased from 16 wt% to 10 wt%, as shown in Figure 1a. The conclusion that the LCST temperature decreases with the increase in PVDF concentration is consistent with our previous study [11]. With the weight ratio of SiO_2_/PVDF increased from 0 to 1:10, the dope solutions M0 to M3 at the same PVDF concentration of 16 wt% showed decreasing cloud points from 50 °C to 37 °C, as shown in Figure 1b. It indicates that introducing hydrophilic SiO_2_ nanoparticles will significantly decrease the LCST of the dope solution.

### 3.2. The Morphology of PVDF/SiO_2_ Composite Membranes

#### 3.2.1. Effect of PVDF Concentration

In general, the introduction of hydrophilic SiO_2_ nanoparticles into the dope solutions decreases the LCST, which is beneficial to an increased ΔT between the coagulation temperature and cloud point of the dope, promoting the TIPS process to form a bi-continuous pore structure without macrovoids. On the contrary, the enhancement of hydrophilicity of the dope solution promoted the exchange of solvent and non-solvent, which is beneficial for the NIPS process and thus long finger-like macrovoid structures. The competition between the above-described factors determines whether the structure of the membrane will be with finger-like macrovoids or bi-continuous pores.

#### 3.2.2. Effect of Weight Ratio of SiO_2_/PVDF on Membrane Morphology

**Figure 2:**
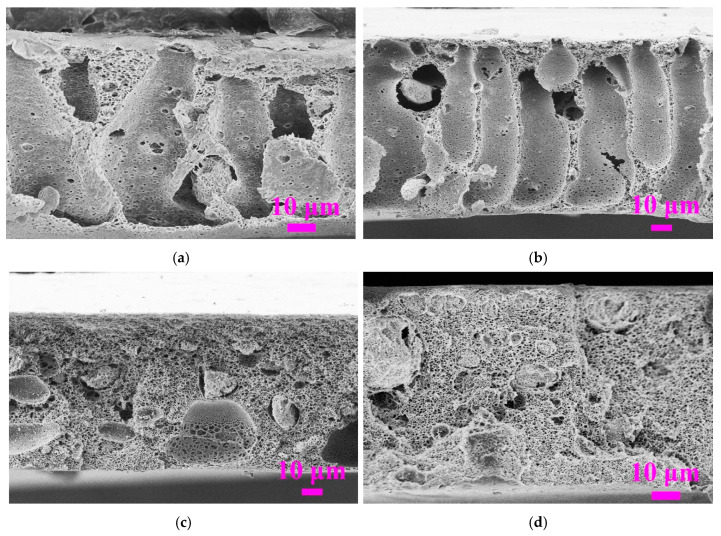
Effect of PVDF concentration on the SEM morphology of PVDF/SiO_2_ composite membranes (the weight ratio of SiO_2_/PVDF = 1:10, coagulation temperature: 90 °C). (**a**) 10% (dope solution M6); (**b**) 12% (dope solution M5); (**c**) 14% (dope solution M4); (**d**) 16% (dope solution M3).

Figure 2 illustrates the cross-sectional morphology of the PVDF/SiO_2_ composite membranes at different PVDF concentrations from 10 wt% to 16 wt% with other parameters kept constant (weight ratio of SiO_2_/PVDF = 1:10 and a coagulation temperature above LCST, i.e., 90 °C). In Figure 2, the cross-sectional SEM images clearly show that with the increase in PVDF concentration from 10 wt% to 16 wt%, the overall membrane structure undergoes a significant transition from having a typical long finger-like macrovoids to bi-continuous porous structure (no macrovoids). At a high coagulation temperature of 90 °C (>LCST), the phase separation process can be explained by the combined NIPS and TIPS (i.e., NTIPS) mechanism according our previous study [11]. The membrane structure transition under different PVDF concentrations can be attributed to the dominant mechanism of either NIPS or TIPS, as the former leads to a finger-like macrovoids structure, whereas the latter leads to a bi-continuous pore structure without macrovoids. At low PVDF concentrations of 10 wt% and 12 wt%, the NIPS mechanism were dominant because of the temperature difference between coagulation and the LCST, i.e., delta T (ΔT) of 37 °C (i.e., 90 °C minus 53 °C) for the 10 wt%, with ΔT of 43 °C (i.e., 90 °C minus 47 °C) for 12 wt% PVDF concentrations, respectively. Such ΔT was not sufficient to induce rapid heat transfer and therefore the TIPS mechanism was less significant, whereas faster exchange rate between solvent and non-solvent led to dominant NIPS effect. On the contrary, at relatively higher PVDF concentrations from 14 wt% to 16 wt%, the TIPS mechanism gradually became dominant because of the higher ΔT between coagulation and LCST of the solution, i.e., 47 °C for 14% and 53 °C for 16%. The higher viscosity of dope solution also suppressed the NIPS process. It is worth noting that the bi-continuous porous structure without macrovoids for the whole membrane cross-section was formed at a much smaller ΔT of 20 °C for the quaternary PVDF/MgCl_2_/PVP/DMAc solution with a low PVDF concentration of 12 wt%, as reported in our previously published work [11]. Although with much higher ΔT, a similar bi-continuous porous pore structure could not be obtained in this new five-component dope solution system with SiO_2_ nanoparticles added. Obviously, embedding hydrophilic SiO_2_ nanoparticles affected the phase separation process in NTIPS, which resulted in obtaining different morphologies. This is because of the faster water diffusion into the nascent membrane that greatly enhanced the solvent and non-solvent exchange rate, leading to a typical macrovoid structure under NIPS.

**Figure 3:**
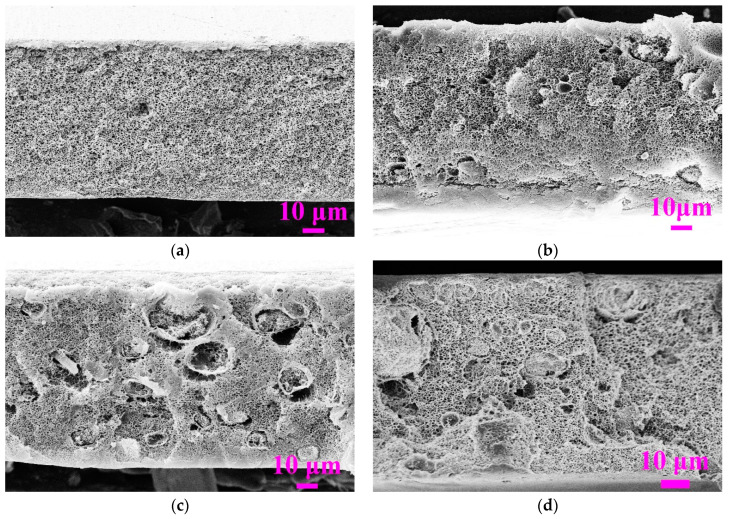
Effect of the weight ratio of SiO_2_/PVDF on the morphology of PVDF/SiO_2_ composite membranes. (**a**) SiO_2_:PVDF = 0 (dope solution M0); (**b**) SiO_2_:PVDF = 1:30 (dope solution M1); (**c**) SiO_2_:PVDF = 1:15 (dope solution M2); (**d**) SiO_2_:PVDF = 1:10 (dope solution M3).

To further investigate the effect of the embedded SiO_2_ nanoparticles on the morphology of PVDF/SiO_2_ composite membranes, membranes with dope solutions at the same PVDF concentration of 16 wt% but with different weight ratios of SiO_2_/PVDF from 0 to 1:10 were made. The dope solution with higher weight ratio of SiO_2_/PVDF was not suitable for casting membrane because of the ultra-high viscosity and difficulty to guarantee the homogeneity of dope solution. Figure 3 illustrated the cross-sectional morphology of the PVDF/SiO_2_ composite membranes with different weight ratios of SiO_2_/PVDF from 0 to 1:10 at the PVDF concentration of 16 wt% and coagulation temperature of 90 °C higher than their LCSTs. It can be found that all of the membranes show bi-continuous pore structure without macrovoids, indicating the dominant effect of TIPS due to the stronger role of ΔT compared to that of the solvent exchange rate, as explained via Figure 2.

#### 3.2.3. Effect of Coagulation Temperature

**Figure 4:**
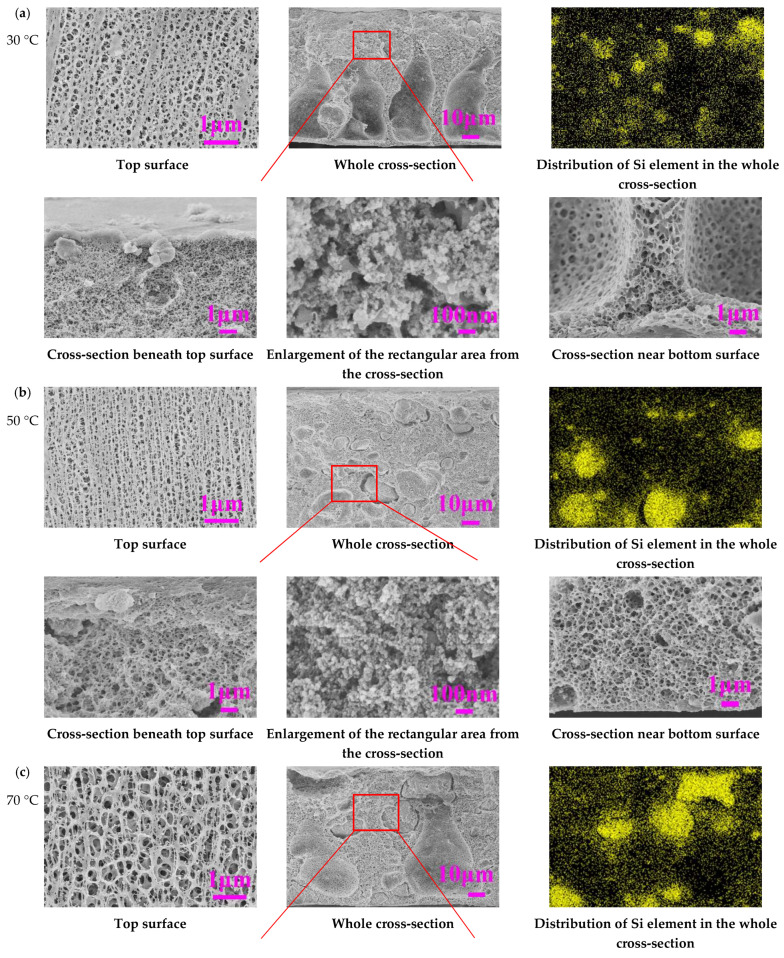

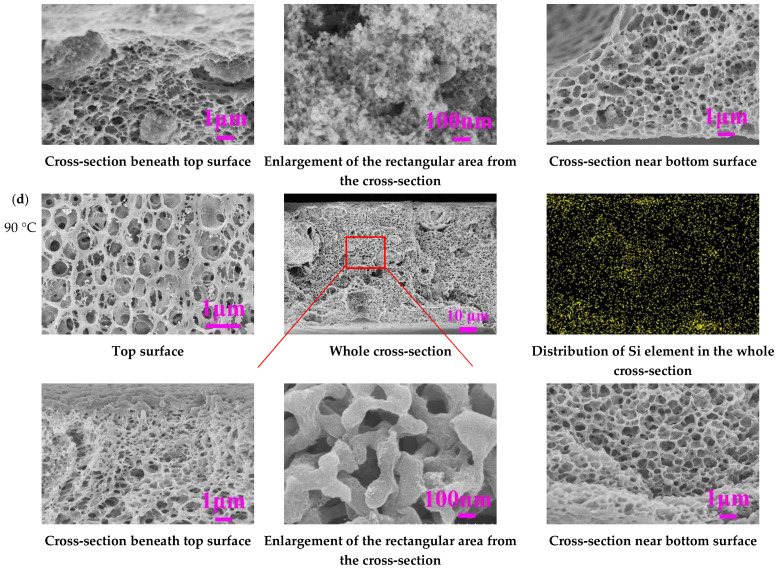
Effect of coagulation temperature on the morphology of PVDF/SiO_2_ composite membranes. (**a**) 30 °C. (**b**) 50 °C. (**c**) 70 °C. (**d**) 90 °C.

Figure 4 illustrates the cross-sectional and top surface morphology of the membranes made at varying coagulation temperatures from 30 °C to 90 °C with constant PVDF concentration of 16 wt% and weight ratio of SiO_2_/PVDF = 1:10, corresponding to membrane samples labelled as T30, T50, T70, and T90, respectively.

In Figure 4, the membrane T30 coagulated at 30 °C exhibited a finger-like macrovoid cross-sectional structure, which was attributed to the typical nucleation growth mechanism in NIPS as the temperatures of both the dope and the coagulant are below the LCST [11,35]. When the coagulation temperature further increased beyond the LCST of 37 °C (Figure 1) and reached 90 °C, the finger-like macrovoids disappeared completely and transformed into a bi-continuous porous structure across the whole cross-section. As discussed in Figure 3, both the NIPS and TIPS mechanisms could play important roles in the membrane formation, depending on the ΔT. At a sufficiently high enough ΔT or coagulation T (e.g., 90 °C), the TIPS dominated and formed desirable bi-continuous pore structure without macrovoids in the whole cross-section. It was also observed that from the cross-sectional structure beneath the top surface, a sponge-like bi-continuous porous structure was observed at all coagulation temperatures, whereas a cellular-like bi-continuous porous structure was formed at the cross-sectional structure near the bottom surface only at the coagulation temperatures above LCST. The sponge-like structure beneath the top surface of the former was due to the NIPS mechanism for T30 and NTIPS mechanism for T50, T70 and T90, whereas the cellular-like structure near the bottom surface of the latter was mainly due to the TIPS mechanism. The results were in good agreement with our previous report of the quaternary PVDF/MgCl_2_/PVP/DMAc solution with LCST characteristics [11].

As shown in Figure 4, all the morphology of the top surface at the above coagulation temperatures exhibited bi-continuous porous structure with high surface porosity, but the surface pore sizes became larger with the increase in the coagulation temperature from 30 °C to 90 °C. The change of top surface structures at an increased coagulation temperature further indicates that the dominant role of TIPS results in more open pore surface and higher surface porosity.

It should be noted that the partial aggregation of SiO_2_ nanoparticles were observed in membranes T30, T50, T70 from the enlarged SEM photos of the rectangular area of the whole cross-section, whereas no observable aggregation of SiO_2_ nanoparticles was found for T90. To further characterize the distribution of SiO_2_ nanoparticles in the PVDF/SiO_2_ composite membranes, SEM-EDX was used and the distribution of Si element in the whole cross-section of these membranes is also shown in Figure 4. It can be observed that the existence of the accumulation of SiO_2_ nanoparticles (yellow dots) were in the cross-section of the membranes T30, T50, T70, whereas Si was dispersed uniformly in the whole cross section of membrane T90 without observable aggregation by the photos of Si elemental analysis.

**Figure 5:**
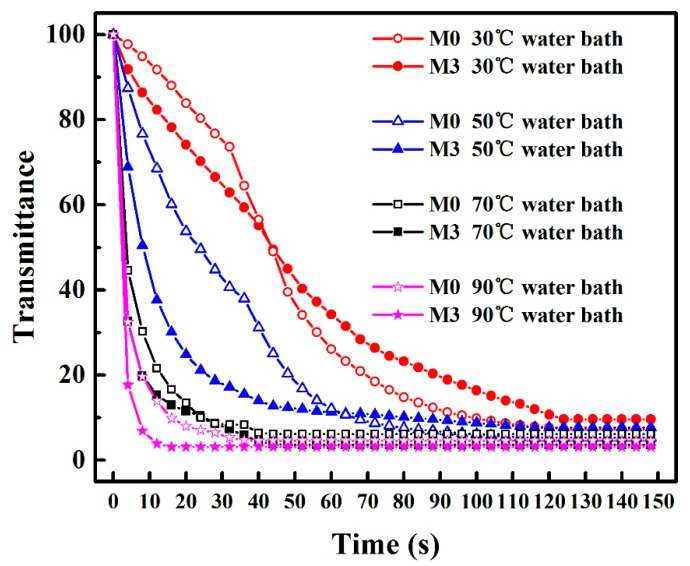
Effect of coagulation temperature on normalized light transmittances of nascent membranes cast with the dope solutions of M0 and M3.

To understand the coagulation kinetics of the nascent membranes cast with the dope solution M3 at different coagulation temperatures, light transmittance experiments were carried out and the results are shown in Figure 5. By varying the coagulation temperature, a series of control membrane sample with no SiO_2_, namely the nascent membranes cast with the dope solution M0, were prepared to serve as the benchmark for the PVDF/SiO_2_ composite membrane cast with dope solution M3 at respective coagulation conditions. As shown in Figure 5, as the coagulation temperature increased from 30 °C to 90 °C, the light transmittance decreases rapidly for both the M0 and M3 groups. The light attenuation rate at the beginning of the formation of the M3 group was faster at the same coagulation temperature compared with that of the M0 group, i.e., showing a steeper decreasing trend at the initial part of the curve. The results indicated that the introduction of hydrophilic SiO_2_ nanoparticles promotes the exchange of solvent and non-solvent, which was explained via the dominant role of the NIPS mechanism in membrane formation. Overall, the phase separation was much delayed at a coagulation temperature below the solution LCSTs. Specifically, for the M3 group it was observed that instantaneous phase separation occurred when the coagulation temperature was above the LCST of 37 °C, i.e., from 50 to 90 °C, resulting in the tendency to form bi-continuous porous cross-section structures without finger-like macrovoids and with a skinless surface, especially at the highest coagulation temperature of 90 °C. For the M0 group, instantaneous phase separation took place at a much higher temperature due to the higher LCST 50 °C, i.e., from 70 to 90 °C. Thus, it was confirmed that above the LCST the dominant mechanism for membrane structure formation was driven by TIPS [11,33].

### 3.3. Membrane Pore Size, Contact Angle, and Mechanical Properties

Other membrane properties such as the porosity, maximum pore radius (*r*_max_), mean pore radius (*r*_m_) and the *r*_max_/*r*_m_, and water contact angle of the membranes made by M3 (with LCST = 37 °C) at different coagulation temperatures from 30 °C to 90 °C are presented in Table 2, the membranes are named correspondingly T30 to T90. It was observed that T30 coagulated below the LCST, exhibiting relatively higher porosity of 84.8% compared with others (<81.4%) coagulated above the LCST. The higher porosity of T30 was mainly due to its finger-like macrovoids structure as shown in Figure 4, whereas the *r*_max_ of the PVDF/SiO_2_ composite membranes was increased from 0.161 μm to 0.203 μm at increasing coagulation temperature from 30 °C to 90 °C. With the increase in the coagulation temperature, the *r*_m_ of the PVDF/SiO_2_ composite membranes had a slight increase from 0.072 μm to 0.101 μm when the temperature was increased from 30 °C to 50 °C and then has a slight decrease to 0.068 μm when the temperature continued to increase to 90 °C. The *r*_max_/*r*_m_ of the membranes at the coagulation temperature from 30 °C to 70 °C was lower than that of the membrane at a coagulation temperature of 90 °C. The larger *r*_max_ and smaller *r*_m_ of the membrane T90 were mainly attributed to the large surface pore size and the typical cellular-like pore formed by TIPS mechanism under the larger ΔT, respectively.

Table 2 shows the contact angle of the membranes fabricated by dope solution M3 at different coagulation temperatures. It can be found that the contact angle of the membranes T50, T70, and T90 coagulated above the LCST obviously decreased compared with that coagulated below the LCST, i.e., T30. Thus, membranes prepared at a higher coagulation temperature exhibited better surface hydrophilicity, particularly at 90 °C, which is consistent with the more homogeneous distribution of the SiO_2_ in the membrane matrix (Figure 4). In addition, the water contact angle of the membranes fabricated by the dope solution M0 with no SiO_2_ at varying coagulation temperatures of 30 °C, 50 °C, 70 °C, and 90 °C was 77 ± 3°, 83 ± 1°, 78 ± 1°, 76 ± 2°, respectively. In general, the membranes fabricated by the dope solution M0 with no SiO_2_ showed a higher water contact angle in the coagulation temperature range (>LCST) compared with those membranes fabricated by the dope solution M3 listed in Table 2.

**Figure 6:**
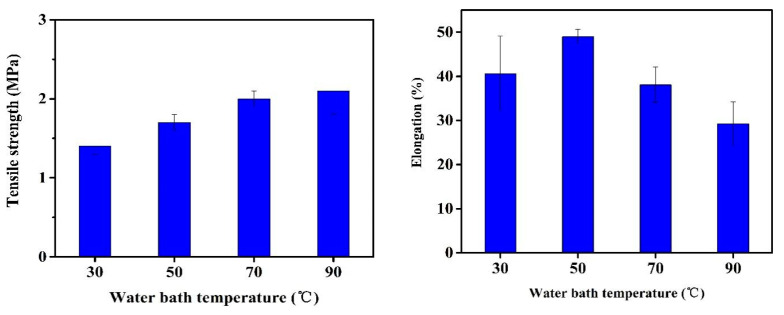
Effect of coagulation temperature on the mechanical properties of the PVDF/SiO_2_ composite membranes.

Figure 6 shows the mechanical properties of the PVDF/SiO_2_ composite membranes fabricated with dope solution M3 at various coagulation temperatures. The tensile strength of the membranes increased from 1.4 MPa to 2.1 MPa when the coagulation temperature increased from 30 °C to 90 °C. The improvement of 50% in tensile strength is mainly attributed to the transformation of the membrane structure from finger-like macrovoids to a bi-continuous porous network, which is consistent with the discussion of Figure 4. This is consistent with observations in literature that the formation of macrovoids leads to weak mechanical strength [36,37]. The elongation at the break of the membranes is first increased from 40.6% to 49% when the coagulation temperature increased from 30 °C to 50 °C, and then decreased to 29.2% with a further increase in temperature to 90 °C. In general, all the membranes had relatively low elongation strength. It could be attributed to the addition of the SiO_2_ nanoparticles that render the membrane more fragile in elongation, which could be related to the compatibility between the nanoparticles and the PVDF matrix and the hindered slippage of polymer chains between nanoparticles [23]. Thus, further improvement on the nanoparticle distribution should be implemented to improve mechanical stability of the membrane. While reflecting on the pore characteristics shown in Table 2, it was observed that membranes with a narrower pore size distribution (i.e., smaller *r*_max_/*r*_m_) were stronger in elongation.

### 3.4. Evaluation of Separation Performance

**Figure 7:**
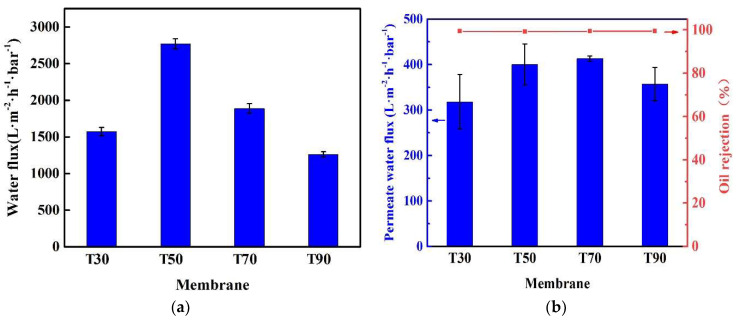

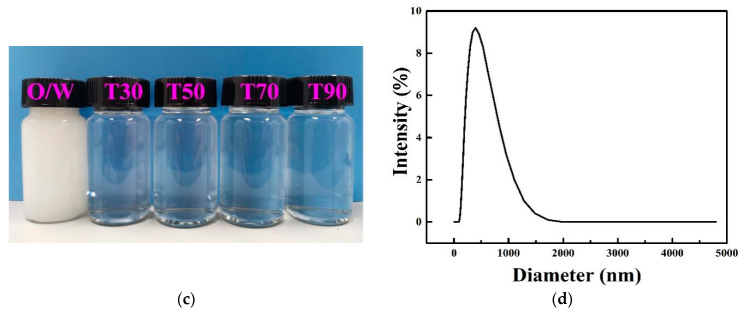
Separation performance of four membranes T30–T90 at varying coagulation temperature: (**a**) pure water flux; (**b**) flux and rejection efficiency with surfactant stabilized emulsion; (**c**) photographs of original emulsion and permeates obtained with various membranes; (**d**) size distribution of oil droplets in surfactant-stabilized oil/water emulsion (dope solution: M3; operation pressure: 20 kPa; ambient temperature, testing period: 60 min).

Figure 7 shows the separation performance of the membrane fabricated with dope solution M3 at different coagulation temperatures via the filtration experiments with pure water and surfactant stabilized oil-in-water emulsion, respectively. The pure water flux of the membranes first increased from 1572 to 2768 L·m^−2^·h^−1^·bar^−1^ when the coagulation temperature increased from 30 °C to 50 °C, and then decreased to 1261 L·m^−2^·h^−1^·bar^−1^ with a further increase in temperature to 90 °C as shown in Figure 7a. The decrease of the pure water flux of membrane T90 coagulated at 90 °C is mainly attributed to the increased mass transfer resistance due to its cellular-like pore structure as shown in Figure 4. Figure 7b shows that the permeation flux of surfactant stabilized oil-in-water emulsion first increased from 318 L·m^−2^·h^−1^·bar^−1^ to 413 L·m^−2^·h^−1^·bar^−1^ when the coagulation temperature increased from 30 °C to 70 °C, and then decreased to 357 L·m^−2^·h^−1^·bar^−1^ with a further increase in temperature to 90 °C. Compared with the pure water flux of these membranes, the permeation flux with the emulsion decreased significantly, which is mainly due to the presence of the oil droplets. The rejection of all the membranes remained high, i.e., between 99.2% and 99.5%. As shown in Figure 7c, all the permeate samples filtrated by the membranes T30 to T90 were transparent. The successful separation of the oil droplets from the emulsion was mainly due to the suitable pore size range of all membranes (Table 2), which is smaller than the mean particle size of oil droplets with a narrow distribution curve (i.e., 0.1–1.7 μm) as shown in Figure 7d.

**Figure 8:**
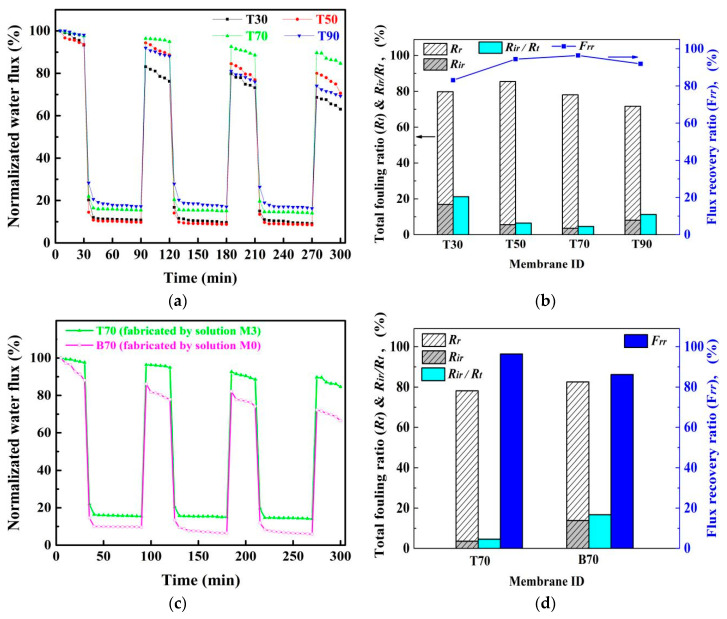
Oil/water emulsion separation: (**a**) normalizated water flux during a 3-cycle filtration of oil/water emulsion by the membranes T30, T50, T70, and T90; (**b**) flux recovery ratio (*F_rr_*), total fouling ratio (*R_t_*), reversible fouling ratio (*R_r_*), irreversible fouling ratio (*R_ir_*), and *R_ir_*/*R_t_* with oil/water emulsion as the foulant after the first cycle; (**c**) comparison of the normalized water flux of T70 with benchmark B70 during a 3-cycle filtration of oil/water emulsion; (**d**) comparison of *F_rr_*, *R_t_*, *R_r_*, *R_ir_*, and *R_ir_*/*R_t_* of T70 and B70 after first cycle.

Membrane fouling is considered the Achilles heel in membrane operation. The main causes for membrane fouling include the adsorption of foulants on the membrane surface and entrapment of foulants in the membrane pores [38]. The main indicators for the fouling tendency of a membrane are reversible fouling and irreversible fouling. Figure 8a showed the normalized fluxes of the composite membranes T30, T50, T70, and T90 in the 3-cycle filtration process, and Figure 8b presents the calculated fouling indicators (*R_t_*, *R_r_*, *R_ir_*, *R_ir_*/*R_t_*, *F_rr_*) based on the first cycle of the results in Figure 8a. It was observed that the normalized water flux of all the membranes significantly decreased when the feed solution was switched from water to the oil/water emulsion as shown in Figure 8a. This was mainly caused by the strong adsorption of oil droplets onto the membrane surface and into the pores [11,39]. The membrane T70 showed a best flux recovery, up to 96.4% of the original value, with just a wash by deionized water in between two filtration cycles, whereas the membrane T30 showed only a flux recovery of 83%. Accordingly, the normalized water flux of the membrane T70 was 85% after the full 3-cycle experiments, which was much higher than that of T30 with only 63% of the original flux, as shown in Figure 8a. It also showed the flux recovery was almost the same for each cycle. Therefore, only the flux recovery ratios (*F_rr_*) and other fouling indicators of the first cycle were representative and figured in Figure 8b. The flux recovery ratios (*F_rr_*) of the membranes coagulated above the LCST of the dope solution M3 (37 °C), i.e., T50, T70 and T90, were higher than the membrane T30 coagulated below the LCST as shown in Figure 8b. The irreversible fouling ratio (*R_ir_*) of the membranes T50, T70, and T90 were significantly lower than that of T30, particularly T70, which exhibited the lowest irreversible fouling ratio (*R_ir_*) value and the lowest ratio of irreversible fouling to total fouling (*R_ir_*/*R_t_*). In addition, Figure 8c,d presents the comparison of the benchmark membrane (named as B70) fabricated by dope solution M0 and the PVDF/SiO_2_ composite membrane fabricated by dope solution M3 at the same coagulation temperature 70 °C (i.e., T70). The ability to maintain a significantly higher flux recovery ratio (*F_rr_*), lower irreversible fouling ratio (*R_ir_*), and the ratio of *R_ir_*/*R_t_* of the T70 was well demonstrated. Thus, the composite membranes made above the LCST condition exhibited excellent anti-fouling performance, mainly attributed to their better hydrophilicity, which reduces the interaction between hydrophobic contaminants such as oil and facilitated mass transfer of water. It was also explained in literature that for a hydrophilic membrane, a hydration layer is formed on the surface due to the electrostatic and hydrogen bonding interactions, eventually leading to fouling reduction [34].

Comparing with several other membranes reported in literature with similar pore size, the as-prepared membrane T70 showed superior separation efficiency in terms of the permeation flux and rejection efficiency, as summarized in Table 3, thus exhibiting promising properties for effectively purifying oil/water emulsions.

## 4. Conclusions

In this study, a new approach was investigated to fabricate high-performance PVDF/SiO_2_ composite membranes via the use of a polymer solution featuring lower critical solution temperature (LCST) characteristics and the non-solvent thermally induced phase separation method (NTIPS). Through morphological observations, synergistic effects between the LCST solution properties and fabrication conditions in NTIPS during the membrane formation process were analyzed to understand the kinetics. It was found that the transition from finger-like macrovoid pores to bi-continuous highly connected pores could be flexibly tuned by increasing the PVDF concentration from 10 to 16 wt%, weight ratio of SiO_2_/PVDF from 1/30 to 1/10 in the dope solution and raising the coagulation temperature to above the LCST of the solution from 50 °C to 90 °C. It was shown that the PVDF/SiO_2_ composite membrane fabricated above the LCST demonstrated much improved hydrophilicity, which resulted in significant enhancement in the anti-fouling properties for oil/water emulsion separation. The permeation flux of the composite membranes with surfactant-stabilized oil-water emulsion was higher than 318 L·m^−2^·h^−1^·bar^−1^, with overall high rejection of 99.2%. The optimal composite membrane T70 (with dope solution M3) was demonstrated via the 3-cycle filtration experiments with a significantly improved flux recovery ratio (*F_rr_*) and minimal reduced irreversible fouling, as compared to its pure PVDF counterpart. Thus, it was successfully demonstrated in this work that facile methods can be developed to regulate the membrane formation kinetics to tailor pore structure for water purification applications.

## Figures and Tables

**Table 1:** Composition of dope solutions.

Dope Solution Code	Composition of Dope Solution (wt%)				
	PVDF	SiO_2_	PVP	DMAc	MgCl_2_
M0	16	0	8	68.0	8
M1	16	0.53	8	67.5	8
M2	16	1.07	8	66.9	8
M3	16	1.6	8	66.4	8
M4	14	1.4	8	68.6	8
M5	12	1.2	8	70.8	8
M6	10	1.0	8	73.0	8

**Table 2:** Porosity, pore size parameters, and contact angle of the membranes fabricated by dope solution M3 at different coagulation temperatures.

Membrane ID	Coagulation Temperature (°C)	Porosity (*ε*, %)	*r*_max_ (μm)	*r*_m_ (μm)	*r*_max_/*r*_m_	Contact Angle (°)
T30	30	84.8 ± 0.5	0.161 ± 0.013	0.072 ± 0.014	2.2	81 ± 2
T50	50	80.6 ± 0.4	0.187 ± 0.014	0.101 ± 0.016	1.9	73 ± 1
T70	70	79.7 ± 1.1	0.186 ± 0.024	0.084 ± 0.021	2.2	74 ± 1
T90	90	81.4 ± 0.7	0.203 ± 0.007	0.068 ± 0.014	3.0	68 ± 2

**Table 3:** Comparison of the as-developed membrane T70 and membranes from selected literature for oily water purification.

Membrane Material	Pore Size (μm)	Type of Oil Emulsion	Oil Droplet Diameter (μm)	Permeate Flux (L·m^−2^·h^−1^·bar^−1^ )	Rejection (%)	Ref.
PVDF/TiO_2_	/	Diesel oil (10 g/L)	1–20	382	99	[40]
Ceramic (modified)	0.11	Soybean oil (0.2 g/L)	1.09	150	97.0	[41]
Ceramic (FATP)	0.6	Engine oil (2 g/L)	5	209	98.7	[42]
PVDF (grafted)	0.45	Soybean oil (100 g/L)	0.7–1.4	10	99	[43]
Polyimide	0.16	Dodecane (5 g/L)	0.63	121	86.6	[44]
PVDF/SiO_2_ (T70)	0.17	Soybean oil (5 g/L)	0.1–1.7	413	99.4	This work

## Data Availability

Not applicable.
